# Imperceptible Somatosensory Single Pulse and Pulse Train Stimulation Oppositely Modulate Mu Rhythm Activity and Perceptual Performance

**DOI:** 10.1093/cercor/bhaa185

**Published:** 2020-08-08

**Authors:** Fivos Iliopoulos, Birol Taskin, Arno Villringer, Till Nierhaus

**Affiliations:** 1 Department of Neurology, Max Planck Institute for Human Cognitive and Brain Sciences, 04103 Leipzig, Germany; 2 MindBrainBody Institute, Berlin School of Mind and Brain, Charité–Universitätsmedizin Berlin and Humboldt-University Berlin, 10099 Berlin, Germany; 3 Department of Education and Psychology, Neurocomputation and Neuroimaging Unit, Freie Universität Berlin, 14195 Berlin, Germany; 4 International Max Planck Research School LIFE, Max Planck Institute for Human Development, 14195 Berlin, Germany

**Keywords:** EEG alpha oscillations, event-related desynchronization/synchronization, inhibition, nonconscious, subliminal

## Abstract

Subliminal stimulation alters conscious perception – a potential mechanism is the modulation of cortical background rhythms especially in the alpha range. Here, in the human somatosensory domain, we assessed effects of subthreshold (imperceptible) electrical finger nerve stimulation – either presented as single pulses or as brief (1 s) 7 Hz pulse trains—on mu-alpha rhythm and perceptual performance. In electroencephalography, subthreshold single pulses transiently (~150–350 ms poststimulus) increased mu activity (event-related synchronization), while, interestingly, subthreshold trains led to prolonged (>1 s) mu desynchronization. In psychophysics, detection of near-threshold target stimuli was consistently reduced when presented together with subthreshold trains (at three delays), whereas for targets paired with subthreshold single pulses detection remained unaffected (30 and 180 ms) or was even elevated (60 ms). Though both imperceptible, single pulses and pulse trains exerted opposite effects on neural signaling and perception. We suggest that the common neural basis is preferential activation of cortical inhibitory interneurons. While the inhibitory impact of a subthreshold single pulse (reflected by mu synchronization) is not psychophysically detectable—rather perception may be facilitated—repetition of the same subthreshold pulse shifts the excitation-inhibition balance toward an inhibitory cortical state (reflected by perceptual impediment) accompanied by mu desynchronization. These differential findings provide novel insights on the notion of alpha activity mediating functional inhibition.

## Introduction

Weak sensory stimuli that escape conscious perception can still modulate brain function and context-dependent behavior. This has been repeatedly demonstrated in behavioral (Reingold and Merikle 1988; Dehaene et al. 2006; Bareither et al. 2014a; Baumgarten et al. 2017) as well as electrophysiological and neuroimaging studies (Kouider and Dehaene 2007; Del Cul et al. 2009; van Gaal et al. 2012; Bareither et al. 2014b). The various mechanisms, however, by which brain functions are modulated by imperceptible stimulation, remain poorly understood.

A potential mediator of the effects emerging from imperceptible stimulation is the modulation of neural oscillations: These are known to underlie fundamental brain functions such as motor control (Miller et al. 2007; Mazaheri et al. 2009; Sauseng et al. 2009), sleep (Massimini et al. 2004), memory (Klimesch 1999), and cognitive performance (Thut and Miniussi 2009; Romei et al. 2010) as well as perception, where alpha power and phase play a crucial role (Busch et al. 2009; Mathewson et al. 2009; Cecere et al. 2015). Beyond the notion of an “idle” operational state (Pfurtscheller et al. 1996), cortical oscillations in the alpha band (~10 Hz) are commonly considered to represent functional inhibition observed on the behavioral level (Klimesch et al. 2007; Jensen and Mazaheri 2010). For instance, high posterior alpha power predicts reduced perceptibility of an upcoming weak visual stimulus (e.g., Ergenoglu et al. 2004; Babiloni et al. 2006; Hanslmayr et al. 2007; van Dijk et al. 2008) and, in attention-related tasks, regions that are not involved in stimulus-related processing exhibit stronger alpha power (Klimesch 2012). Furthermore, it has been shown that low prestimulus alpha power also translates into higher visually evoked blood oxygenation level-dependent (BOLD) signal (Becker et al. 2011) and that also the phase of alpha rhythm influences evoked BOLD signal (Scheeringa et al. 2011). Finally, imperceptible stimulation itself has been shown to modulate cortical oscillatory activity (Balconi and Ferrari 2012; Bareither et al. 2014b; Nierhaus et al. 2015; Simon and Mukamel 2016; Forschack et al. 2017; Ten Oever et al. 2017).

For the somatosensory system, we face a similar set of findings. Amplitude of sensorimotor alpha rhythm, that is, mu rhythm, influences perception of somatosensory stimuli (Linkenkaer-Hansen et al. 2004; Zhang and Ding 2010; Weisz et al. 2014) and modulates late evoked potentials that are related to perceptual processing (Reinacher et al. 2009; Schubert et al. 2009). These findings already indicate that any external intervention, which modulates background alpha/mu rhythm, could alter somatosensory perception of an upcoming stimulus. Interestingly, we have recently shown that single subthreshold pulses lead to a transient mu rhythm increase (synchronization) over the contralateral pericentral region (Nierhaus et al. 2015). Previously, we had found that imperceptible pulse train stimulation impaired detection of intermingled near-threshold target stimuli and, furthermore, led to a negative BOLD response in contralateral primary somatosensory cortex (cS1; Blankenburg et al. 2003; Taskin et al. 2008), which in turn is probably related to cortical inhibition as shown in several other systems (Hamzei et al. 2002; Hlushchuk and Hari 2006; Shmuel et al. 2006).

Bringing these findings together, we assume that external stimulation, which modulates background alpha rhythm, would also alter perceptual performance. We hypothesize that changes in mu rhythm synchrony following subthreshold stimulation can be related to a psychophysically observable effect and thus might represent the underlying cortical mechanism. In this context, it was unclear to us whether the functional inhibition we reported for subthreshold train stimulation is simply the accumulation of single pulse repetition. In order to directly compare the two subthreshold stimulation conditions, that is, single pulses versus pulse trains, we performed a systematic study. Using electroencephalography (EEG), we investigated somatosensory evoked potentials (SEPs) and spectral alterations following subthreshold electrical finger nerve stimulation. In a complementary psychophysical approach, we measured perceptibility of target pulses (TPs) when combined with either subthreshold single pulses or pulse trains. By testing TPs delivered at different characteristic latencies—with respect either to the kinetics of cortical inhibition (Swadlow and Gusev 2000) or to the previously confirmed subthreshold effect on mu rhythm (Nierhaus et al. 2015)—we compare the effect of subthreshold pulses on the detection of targets that are delivered either after a preceding single pulse or delivered together with a subthreshold train. Specifically, we hypothesized that subthreshold trains would also increase mu-alpha synchronization—due to repetition possibly even stronger than a single pulse—and that both single pulses and brief trains would induce functional inhibition.

## Materials and Methods

### Subjects

We performed one EEG and six psychophysical experiments on a total of 158 participants (female, 84; male, 74; age range, 18–38 years). For EEG recordings, 40 healthy volunteers were recruited (mean age and standard deviation [SD] 28.6±2.8 years). Psychophysical measurements were performed independently in six separate experiments: A1: *n* = 20, 25.3 ± 2.7 years; A2: *n* = 21, 26.1 ± 2.9 years; A3: *n* = 19, 27.0 ± 2.6 years; B1: *n* = 21, 25.1 ± 2.3 years; B2: *n* = 19, 26.0 ± 2.8 years; B3: *n* = 18, 26.0 ± 2.8 years. All subjects were right handed with a mean laterality score of 89.0 ± 12.9 SD (within a range of −100 to +100, i.e., fully left and right handed, respectively, according to Edinburgh Handedness Inventory; Oldfield 1971). None of the subjects had a history of any neurological/psychiatric disorder or medication. Experiments were performed in accordance with the principles of the Declaration of Helsinki. Prior to participation, all volunteers gave written informed consent to participate in the experiment. This study was approved by the ethics committee of the University of Leipzig, Germany (Nr. 462/15-ek).

### Electrical Finger Stimulation

**Figure 1:**
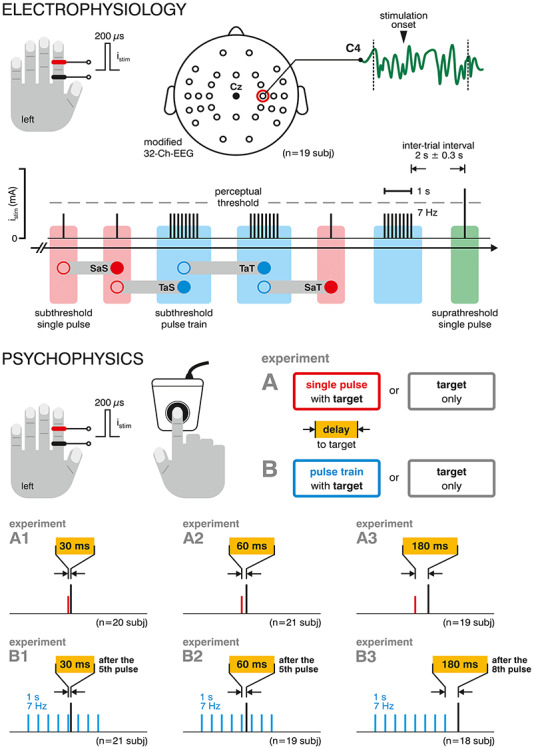
Schematic overview of experimental procedures. Electrophysiology: acquisition of an extended 32-channel EEG (40 subjects) during electrical nerve stimulation of the left index finger with subthreshold single current pulses (red), brief (1 s) subthreshold current pulse trains (eight pulses at 7 Hz, blue), as well as—to maintain attentional level—suprathreshold single pulses (*green*) in a pseudo-randomized order. Each subthreshold stimulation epoch was sorted offline according its pre-trial history (labeled, e.g., “single after train”, SaT), that is, whether it was presented after a subthreshold single pulse or pulse train. C4 electrode signal time courses underwent preprocessing, segmentation, and averaging (SEPs) as well as TFA. Psychophysics: Subthreshold single pulse or pulse train (1 s at 7 Hz) stimulation was combined with presentation of near-threshold TPs at different delays (30 ms, 60 ms, and 180 ms), resulting in six different conditions that were compared to the control condition (i.e., TP presentation without any subthreshold stimulation) in separate experiments (A1 to A3 and B1 to B3). In experiments B4 and B5, the TP was delivered after the fifth subthreshold pulse of the train (i.e., embedded in the train). In experiment B3, the target was delivered 180 ms after the last subthreshold pulse. The paradigm drawings are displayed out of scale for illustration purposes. Subjects’ responses were recorded (button press) in a simple detection task.

For somatosensory stimulation, single monopolar square-wave current pulses (duration 200 μs) were delivered to the sensory nerves of the left index finger via steel-wire ring electrodes placed on the middle and proximal phalanx with the cathode located proximally (Fig. 1); the constant-current stimulator (DS series, Digitimer) was controlled by routines written in Matlab (Version R2017a, MathWorks) and Presentation (Neurobehavioral Systems). Prior to an EEG or psychophysics experiment, individual absolute detection threshold and respective stimulation amplitudes were assessed as previously described in detail (Nierhaus et al. 2015; Forschack et al. 2017). The absolute detection threshold is considered as the lowest current intensity for a continuous 7 Hz pulse train (0.1 mA steps) at which a subject was still able to report a sensation (method of constant stimuli). The current intensity for subthreshold single pulse and pulse train stimulation was set ~ 15% below the absolute detection threshold to ensure it is indeed reliably imperceptible throughout the entire experiment. This procedure was confirmed to provide undetectable stimulus intensities as we have previously demonstrated using a one-alternative forced choice detection task (Forschack et al. 2017).

### E‌EG Acquisition

Thirty-two-channel EEGs (modified 10–20 system; reference FPz) were acquired with the BrainAmp amplifier/AD converter system and respective recording software (0.015–1 kHz band pass filter; sampling frequency, 5 kHz; Brain Products). The stimulation paradigm comprised two conditions where subthreshold single pulses and subthreshold pulse trains at 7 Hz (repetition of eight pulses; duration, 1 s) were delivered to the left index finger in a pseudo-randomized order (mean interstimulus interval, 2 s; jitter, ±300 ms). The EEG paradigm contained 360 trials per either condition and was divided in 12 blocks (duration ~ 2.5 min) separated by brief pauses. After the sixth and the last block, the initially determined absolute detection thresholds were checked for potential drifts. Additionally, rarely interspersed low-intensity suprathreshold single pulses (four per block) on the left and right index finger were applied; subjects were instructed to count and report perceived pulses in each inter-block pause: with this instruction, we aimed to retain potential attentional fluctuation acceptably low and induced a level of attention comparable to the setting in the behavioral experiments. Furthermore, as EEG effects of subthreshold stimulation are subtle and therefore difficult to detect, the acquisition of suprathreshold SEPs serves as a kind of “internal calibration” to exclude systematic errors in the data acquisition or analysis overall. Electrode impedances were <5 kΩ throughout all measurements. Following our previous approach (Nierhaus et al. 2015; Forschack et al. 2017), we preselected electrode C4 (i.e., over pericentral region contralateral to stimulation site) for further analyses and statistical testing.

### E‌EG Data Analyses

EEG data were analyzed off-line using custom-built Matlab scripts. Data were digitally filtered using a standard third order bandpass Butterworth filter (low cutoff frequency, 1 Hz; high cutoff frequency, 45 Hz). After downsampling to 500 Hz and concatenating all blocks, the data set of each subject underwent an independent component analysis (ICA) to remove sources of ocular artifacts (Li et al. 2006).

#### SEPs

An EEG segmentation analysis was performed on data obtained from electrode C4 as it captures signaling from the hand in area cS1/pericentral region most closely. For subthreshold stimulation, this is indicated by the P60 component as we have previously shown (Nierhaus et al. 2015; Forschack et al. 2017). Epochs were defined ranging from −200 to 2400 ms for subthreshold train stimulation trials. SEPs were obtained by averaging all trials or by averaging a subset of trials depending on the pre-trial history. To evaluate the P60 component, time points from 55 to 65 ms were averaged and compared to baseline (−200 to 0 ms) or between conditions in a paired *t*-test.

#### Rolandic Rhythms

Since Rolandic rhythms can be hidden under predominating occipital alpha activity, a preselection of “central” ICA components was performed before trial segmentation (Nierhaus et al. 2015; Forschack et al. 2017) in order to isolate and include only sources of Rolandic oscillatory activity that are related to somatosensory processing. Rolandic rhythms are characterized by a central localization and a power spectrum that exhibits two characteristic peaks, at alpha (7–14 Hz) and beta (15–29 Hz) frequency bands, respectively (Jones et al. 2009), which both desynchronize after suprathreshold stimulation. Based on this operational definition to identify Rolandic rhythms, for each subject only those components obtained by ICA were selected, for which the following criteria applied: 1) central topography, 2) the two respective peaks (alpha and beta) in the frequency spectrum, and 3) a desynchronization episode after suprathreshold stimulation. Consequently, two to seven (mean, 3 ± 1 SD) independent components were selected in each subject’s data set, which were back-projected to the electrode space and segmented to subthreshold epochs as defined above. To allow for a time-resolved frequency analysis, a wavelet analysis for the frequencies from 4 to 30 Hz in 1 Hz increments was performed on single trial epochs using a five-cycle long wavelet. The resulting time-frequency data were finally averaged over trials of interest according to the corresponding stimulation condition. A 200 ms prestimulus epoch (−200 ms to 0 ms) was defined as baseline. For the time-frequency data, we used a nonparametrical cluster-based permutation test (Maris and Oostenveld 2007) with 1000 iterations: at the subject level, in each iteration, we permuted the mapping either between post-onset data points of the two conditions (“SaS” vs. “SaT”; Fig. 3B) or between post-onset data points and baseline values (Fig. 4A) and used a paired *t*-test at the group level to test each data point for significance (i.e., whether the signs were consistent). Then, we assessed the sum of *t*-values within largest contiguous cluster of significant time-frequency points (threshold *P* < 0.05), resulting in a distribution of *t*-value sums expected under the null hypothesis. Clusters in the observed data exceeding the family-wise error-corrected threshold (pFWE < 0.05) were considered to be statistically significant.

### Psychophysical Experiments

In a simple detection task, subthreshold stimulation was combined with near-threshold stimulation (target) at one of three different delays with respect to the concomitant subthreshold event, i.e., either a single subthreshold pulse or a 7 Hz pulse train of eight pulses and 1 s duration (Fig. 1): In experiments A1 to A3, subthreshold single pulse stimulation was paired with a succeeding TP at an interpulse delay of 30, 60 (characteristic inhibition decay latencies; Swadlow and Gusev, 2000), and 180 ms (subthreshold effect on EEG rhythm; Nierhaus et al. 2015); for concomitant subthreshold pulse train stimulation, the TP was applied either within the train (trials resembling continuous subthreshold stimulation; Taskin et al. 2008) after the fifth pulse at a delay of 30 or 60 ms (experiments B1 and B2) or after the entire pulse train, with a delay of 180 ms (experiment B3). Each of these conditions was tested independently in separate experiments (Fig. 1; experiments A1 to A3 and B1 to B3) on different groups of subjects. Prior to an experiment, the subthreshold stimulation intensity was determined following the procedure described above. Each experimental block started with a staircase procedure to determine the individual 50% detection threshold, by which five linearly increasing near-threshold intensities were set as TP intensities. A single experiment consisted of three blocks (~9 min duration each) and comprised two types of trials: Presentation of TP only (control) and TP with concomitant subthreshold stimulation; in a single block, each TP intensity was repeated 32 times, while in half of the trials (i.e., 16 per intensity), the TPs were accompanied by subthreshold stimulation. Subjects were instructed to press a button (right index finger) as fast as possible whenever they detected a stimulus. This simple detection task allows no comparison between changes in sensitivity versus criterion (according to signal detection theory [SDT]). We nevertheless chose this design in order to minimize attentional shifts and visuo-cognitive interaction by monitor-displayed instructions that would likely “overshadow” the weak effects elicited by the subthreshold stimuli in the EEG experiment. To overcome this limitation, a much longer EEG experiment would have been required to obtain enough signal to noise for these small signals. Such a prolonged paradigm, however, would be of nonfeasible duration with additional unpredictable time-dependent contaminative effects (e.g., habituation or attentional shifts). Although this design ensures certain similarity of the behavioral and EEG experimental design, the comparison of the resulting effects induced by subthreshold stimulation has to be interpreted with caution due to the still existing discrepancies. Catch trials containing only subthreshold stimulation (16 per block) were additionally included to control for the stimulus’ imperceptibility; a subject’s data would be discarded if a button response was given to more than one catch trial (in a total of 354 recorded blocks this occurred only in two blocks for different subjects). For statistical analysis, in each of the six psychophysical experiments, the individual detection rates for the test and control conditions were averaged and compared using a paired *t*-test. Changes in detection performance are reported as relative changes to the respective control condition (trials containing only TPs), that is, (rate_condition_ − rate_control_)/rate_control_.

## Results

### Subthreshold Single Pulse Stimulation: SEPs and TFA

**Figure 2:**
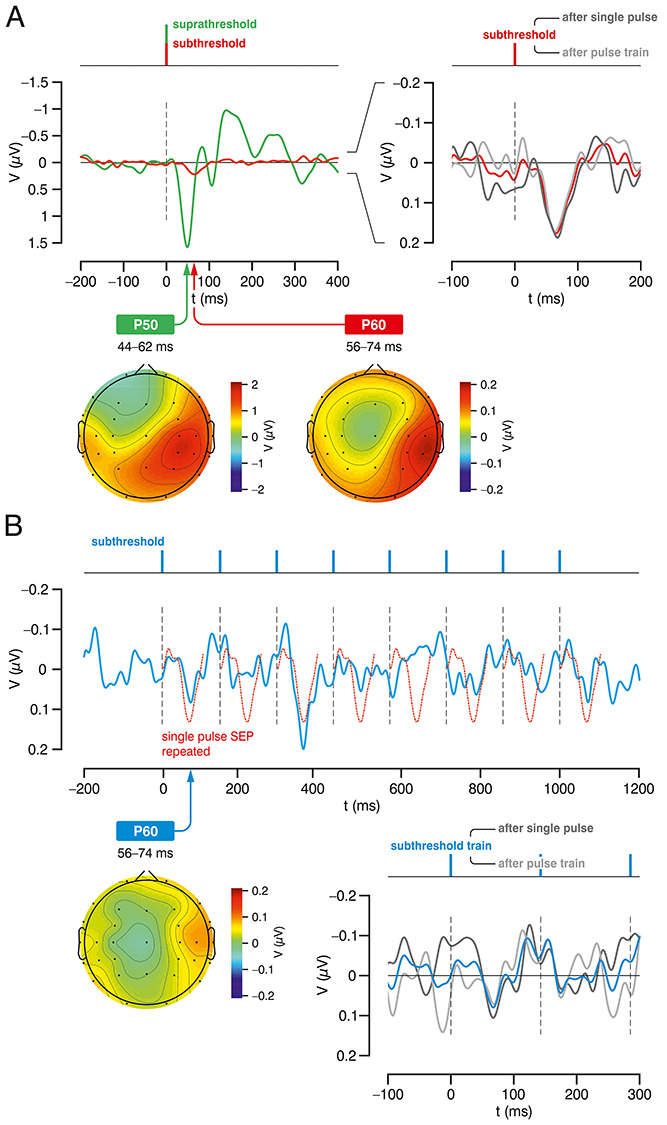
SEPs. (*A*) Grand average SEPs (40 subjects) in response to single pulse stimulation on the left index finger. Left: subthreshold (red) SEP confirming the characteristic P60 component; suprathreshold SEP (green). Right: SEPs for subthreshold stimulation sorted according pre-trial history (SaS and SaT; dark and light gray, respectively), both comprising a P60 component (no significant difference). Below: topographic maps of the P50 and P60 component for supra- and subthreshold single pulse stimulation, respectively. (*B*) Grand average (blue) for subthreshold train stimulation with presumable initial pulse-related component but lacking consecutive train-driven synchronicity (dotted red: single pulse-SEP shown in Fig. 2A drawn repetitively as a ‘‘pseudo-phase-locked’’ response to each pulse of the train for comparison); bottom right: grand average SEPs for subthreshold train stimulation (initial part) sorted according to pre-trial history (train, TaS, and TaT; blue, dark, and light gray, respectively); bottom left: topographic map of the initial P60 component in response to subthreshold train stimulation.

**Figure 3:**
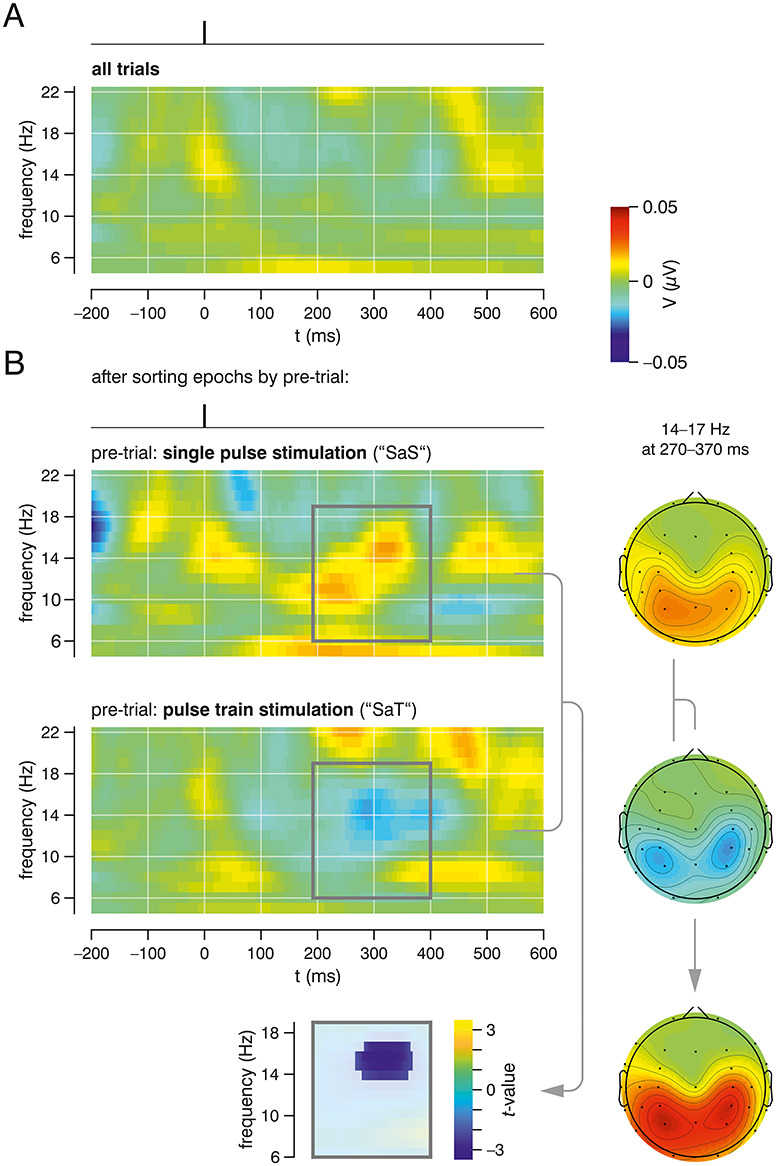
TFA for subthreshold single pulse stimulation. (*A*) Average from all trials does not show any significant change in EEG frequency spectrum. (*B*) Averages from respective trials after pre-trial sorting: “single pulse after single pulse” (SaS, upper panel) and “single pulse after pulse train” (SaT, lower panel). Bottom: Nonparametric statistical analysis (contrasting SaS vs. SaT) reveals a significant cluster according to the expected rhythm change at ~ 200 ms poststimulus. The window for statistical comparison was chosen a priori based on previous findings (Nierhaus et al. 2015). Right column: topographic maps of the significant cluster (14–17 Hz, 270–370 ms; as determined by the above-mentioned nonparametric test) for SaS and SaT conditions, as well as their difference (for illustration purposes only, without statistical testing).

Subthreshold single pulse stimulation elicited a weak (in comparison to the suprathreshold pulse; Fig. 2A) voltage response (grand average) containing a single discernible (i.e., statistically significant) positive component at a peak time ~60 ms, the P60 [Fig. 2A; mean amplitude of the 55 to 65 ms poststimulus interval vs. baseline, *t*(39) = −2.7399, *P* = 0.0092], thus confirming our recent finding (Nierhaus et al. 2015; Forschack et al. 2017). Consequently, we would have also expected to find an event-related synchronization in the mu-alpha band as described in our above-mentioned studies. However, the absence of a similar spectral alteration in the present EEG data (Fig. 3A) as well as previous findings concerning the impact of past trial history for behavioral data (Thiel et al. 2014) enforced the assumption that processing in cS1 associated with the preceding trial (i.e., subthreshold single pulse or pulse train stimulation) might still interfere with the cortical response to the very succeeding stimulation event. To control for a potential history trial-related hysteresis effect, we sorted EEG epochs with regard to the previous trial condition “single pulse” or “pulse train” stimulation, which were labeled “SaS” (single pulse after single pulse) and “SaT” (single pulse after pulse train), respectively. The resulting grand SEPs both exhibited a respective component within the P60 window [Fig. 2; SaS: *t*(39) = −2.0434, *P* = 0.0478; SaT: *t*(39) = −2.143, *P* = 0.0384], being not significantly different [SaS vs. SaT: *t*(39) = 0.0484, *P* > 0.9].

When sorting the single pulse time-frequency analyses (TFAs) according to the past trial, we observe differential changes in the alpha frequency band: When averaging only the events following single pulse stimulation (SaS; Fig. 3B), a synchronization pattern emerges ~ 200 ms after stimulus onset and continues up to > 300 ms (similar to what we have initially expected based on our previous studies; Nierhaus et al. 2015; Forschack et al. 2017), whereas trials following subthreshold train stimulation (SaT; Fig. 3B) are associated with a poststimulus desynchronization from ~ 250 to > 300 ms. While these desynchronization/synchronization patterns are not significant versus baseline alpha power, the contrast between the sorted TFA conditions (SaS versus SaT) shows a significant cluster (FEW-corrected *P* < 0.041; Fig 3B, right panel). The time-frequency window for the statistical comparison was set according to our a priori hypothesis derived from our previous findings (Nierhaus et al. 2015).

### Subthreshold Train Pulse Stimulation: SEPs and TFA

In the grand average SEP obtained for subthreshold pulse train stimulation, the initial P60 component of the first intra-train pulse was not significant compared to baseline [Fig. 2B; *t*(39) = −1.234, *P* > 0.2], yet still shows a similar shape and matching latency as for the subthreshold single pulse [Fig. 2B; red overlay, no significant amplitude difference, *t*(39) = −1.489, *P* > 0.14]. For the subsequent pulses, no clear latency pattern was observed (except for a biphasic potential between the third and fourth pulse of unclear relevance). After pre-trial sorting, we find that trains following single pulses (TaS) exhibit a significant initial P60 deflection [precomponent time segment vs. P60, i.e., 38–42 ms vs. 66–70 ms; *t*(39) = −2.313; *P* = 0.0261]; however, for trains after trains (TaT), there is no discernible P60 [precomponent time segment vs. P60, *t*(39) = −0.729; *P* > 0.4]. It seems that strong baseline fluctuations were critical to significantly detect the (subtle) initial P60 component for train stimulation.

**Figure 4:**
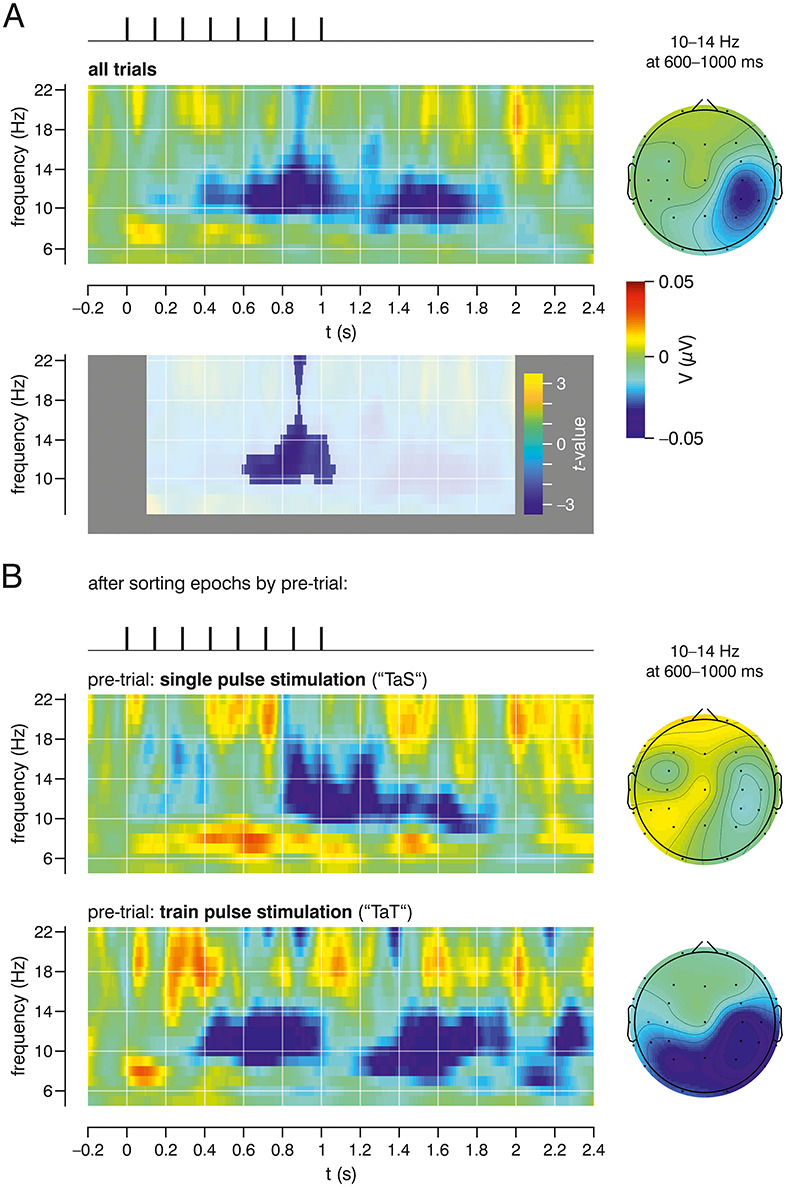
TFA for subthreshold pulse train stimulation. (*A*) Average from all subthreshold train stimulations (unsorted trials; upper panel). In the nonparametric statistical analysis (comparison of the 100 ms–2 s window against baseline, i.e., –200 ms to 0 ms; lower panel), a single significant cluster (inlay) is specified. (*B*) TFAs for subthreshold pulse train stimulation after pre-trial sorting: “train after single pulse” (TaS, upper panel) and “train after train” (TaT, lower panel). Both show a decrease in the mu-alpha range emerging during train stimulation and lasting for ~ 1 s after (not significant in cluster analysis). Right: Topographic maps of the significant cluster (10–14 Hz, 600–1000 ms; as determined by the nonparametric test in *A*) for all trials and after pre-trial sorting (TaS and TaT).

Subthreshold train stimulation induced a marked desynchronization of ongoing mu-alpha activity (Fig. 4A), being statistically significant (lower panel; nonparametrical cluster FWE-corrected *P* = 0.044); desynchronization started to evolve ~ 100 ms after stimulation onset and further increased with succeeding pulses. During train stimulation, for a brief episode, desynchronization appeared also in the beta frequency band. The alpha desynchronization continued beyond the end of the pulse train for ~ 1 s (i.e., persists after stimulation offset). Thereafter, a subsequent short increase of synchrony in the beta frequency band can be observed (“beta rebound”; statistically not significant). After pre-trial sorting, respective TFAs both showed mu rhythm desynchronization; however, for successive train stimulation (TaT), desynchronization emerged earlier and continued for a considerably longer time (>2 s) as compared for the single pulse pre-trial condition (TaS; Fig. 4B). The maintenance of the desynchronized state is also supported by the desynchronization induced by single pulse stimuli that follow pulse trains (SaT; Fig. 3B), that is, although subthreshold single pulse stimulation is associated with an increase in the amplitude of mu-alpha (Fig. 3B), a pulse train persistently shifts network dynamics so that a following single pulse is enough to reinduce desynchronization. In other words, the dominant persisting alpha desynchronization induced by the repetitive stimulation is responsible for the absence of mu-alpha synchrony when averaging over all single pulse trials (Fig. 3A).

**Figure 5:**
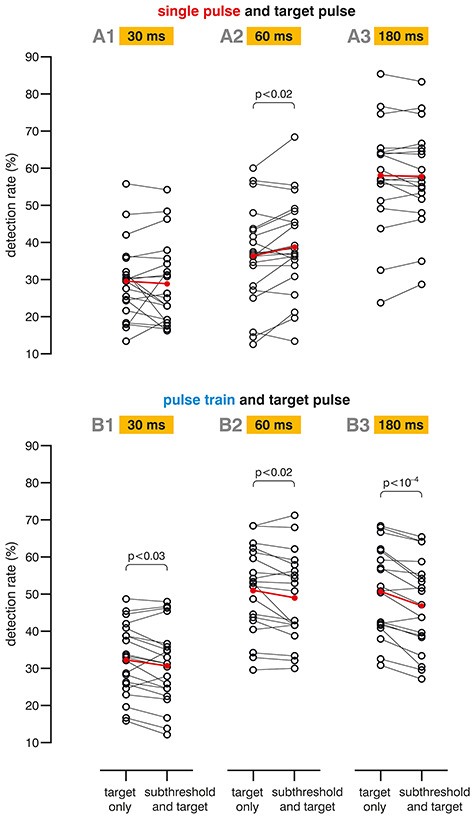
Psychophysical performance. Detection rates obtained in six separate experiments (A1 to A3 and B1 to B3; as described in Fig. 1). Upper panel: Subthreshold single pulse stimulation was associated with an increase of mean detection rate by 6.5% (relative change, i.e., as compared to the target only condition) for a delay of 60 ms between near-threshold TP and preceding subthreshold single pulse; for the other delays (30 and 180 ms), no significant change was found. Lower panel: Subthreshold pulse train stimulation induced a significant decrease in mean TP detection rates for all tested delays, by 5.0%, 3.9%, and 7.7% (relative changes) for a delay of 30, 60 (after the fifth pulse of the train, respectively), and 180 ms (after end of the train).

### Psychophysics: Impact of Subthreshold Stimulation on TP Detection

We complemented the electrophysiological study with psychophysical experiments where we investigated changes in perceptibility of near-threshold TPs when paired with a subthreshold single pulse or a subthreshold pulse train at different delays (Fig. 1). For a delay of 60 ms, subthreshold single pulse stimulation led to a significant increase of mean TP detection rate by 6.5% [Fig. 5; relative to target only; *t*(20) = 2.6545, *P* = 0.0152]. For the delays 30 and 180 ms, no significant effect on TP detection was found [*t*(19) = −0.5106, *P* > 0.615 and *t*(18) = −0.7159, *P* > 0.482, respectively]. In contrast, with concomitant subthreshold pulse train stimulation, mean TP detection rates were reduced for all delays tested: by 5.0% for 30 ms [*t*(20) = −2.4025, *P* = 0.0261], by 3.9% for 60 ms (*t*(18) = −2.5993, *P* = 0.0181), and by 7.7% for 180 ms [*t*(17) = 5.1942, *P* < 10^−4^].

## Discussion

We performed somatosensory subthreshold (i.e., imperceptible) single pulse and pulse train stimulation to look for its EEG signatures and examined its impact on perceptibility of near-threshold TPs. The comparison of subthreshold single pulse stimulation between conditions of different preceding stimulus (SAS vs. SAT) disclosed a transient pattern of increased mu-alpha synchronization for subthreshold single pulses following single pulses (~150 to ~300 ms after stimulation), similar to what we expected based on our previous studies. Conversely and being a novel finding, repetitive subthreshold stimulation led to a prolonged mu-alpha desynchronization evolving ~ 400 ms after train onset and outlasting the train at least 800 ms. Interestingly, the modulatory effect of subthreshold train stimulation even exceeded the period of desynchronization, since mu-alpha activity of subsequent trials was still affected (e.g., mu-alpha synchronization was absent when the single pulse followed a previous train stimulation). In psychophysics, subthreshold train stimulation (1 s at 7 Hz) decreased perceptibility of near-threshold TPs at all tested delays (30, 60, and 180 ms). In contrast, single subthreshold pulses either enhanced detection of TPs (at 60 ms delay) or had no significant effect (at delays of 30 and 180 ms, respectively).

Single subthreshold pulse stimulation evoked a P60 component (together with mu-alpha synchronization) confirming central processing of subthreshold stimulation (Nierhaus et al. 2015; Forschack et al. 2017). For subthreshold train stimulation, a P60 deflection related to the initial pulse of the train was detected while subsequent phase- or stimulus-locked deflections were missing, that is, no stimulus-driven oscillations in terms of rhythm entrainment were observed. Notably, the P60m component of somatosensory evoked fields in response to median nerve stimulation has previously been shown to vanish at a repetition time of 150 ms (i.e., at ~ 7 Hz, though this occurred under steady-state conditions; Wikström et al. 1996).

In previous studies, we already demonstrated that subthreshold electrical finger nerve stimulation elicited a negative BOLD response in cS1 and moreover diminished the positive BOLD response to suprathreshold stimulation (Blankenburg et al. 2003; Taskin et al. 2008). A negative BOLD signal change from baseline (“fMRI deactivation”) is assumed to mirror a suppressed state of cortical activity and was repeatedly attributed to indirectly reflect synaptic inhibition in different systems (e.g., Gusnard and Raichle 2001; Hamzei et al. 2002; Hlushchuk and Hari 2006; Shmuel et al. 2006). Furthermore, we previously reported an impeding effect of sustained subthreshold train stimulation (20 s) on TP detection (Blankenburg et al. 2003; Taskin et al. 2008). Following these previous studies, we here chose again the “simple detection task” because the subtle behavioral and EEG changes following subthreshold stimulation would be difficult to detect when superimposed by large attention- and visuomotor-related effects induced in an SDT-based design. We now show that brief subthreshold pulse trains of 1 s duration are already sufficient to elicit this functional inhibition. We regard the low intensity of subthreshold stimulation to be crucially responsible for the inhibitory effects we observe. This is strongly supported by various in vivo and in vitro experiments on thalamocortically mediated feedforward inhibition in cS1: Inhibitory interneurons of somatosensory barrel cortex were found to have considerably lower excitation thresholds than principal neurons (Swadlow 1995; Gil and Amitai 1996; Swadlow and Gusev 2000). Thus, the weak subthreshold stimulation in our experimental design is more likely to selectively activate a larger population of cortical inhibitory interneurons. As for the kinetics of synaptic inhibition, inhibitory postsynaptic potentials (IPSPs) in the targeted pyramidal cells (elicited disynaptically via local inhibitory interneurons) exhibited a peak at ~ 30 ms and decayed within another 30 ms (Swadlow and Gusev 2000). Accordingly, in the psychophysical experiments, we time-locked TPs to subthreshold stimulation at 30 and 60 ms. Driven by our finding of mu-alpha synchronization (as outlined below putatively resembling lowered cortical excitability), we additionally tested a delay at which we would expect interaction of TP-related processing with oscillatory activity induced by preceding subthreshold stimulation (maximum at ~ 200 ms; by choosing 180 ms, we have taken into account a latency of ~ 20 ms until peripheral stimulation reaches cS1). Consequently, for the single pulse condition, we anticipated target detection would be impaired at the maximum of the IPSP (30 ms) as well as during the mu-alpha increase (180 ms), whereas at the low state of inhibition (60 ms, late decay phase) detection would remain unchanged. Surprisingly, no detection impairment was seen for the 30 and 180 ms delays, while at 60 ms a facilitation occurred. Possibly, time locking the TP to the IPSP decay phase results in noise reduction as compared to random target presentation in the control condition. Another critical parameter for the paired pulse detection might be the rhythmical modulation of perception driven by the subthreshold stimulus. While the model of perceptual cycles was not developed based on mere detection but on time discrimination tasks (Baumgarten et al. 2017), the rhythmical modulation of discreet cycles in the beta band (13–18 Hz) might also contribute to the selective detection enhancement of TPs falling in the respective time window (55–77 ms). This is also in line with the model we discussed above, according to which the duration of each perceptual cycle is defined at the cellular level (decay of inhibition). A single subthreshold pulse is probably not sufficient to elicit a psychophysically measurable inhibitory effect at the other delays. The claim that synaptic inhibition is indeed enhanced by subthreshold stimulation is still supported by the impaired perceptual performance due to repetitive stimulation (train).

How do subthreshold stimulation and inhibition relate to oscillatory activity in cS1? Fundamental determinants of mu rhythm generation and modulation are: 1) thalamocortical excitation of cortical pyramidal cells and cortical feedback within the thalamocortical loop (the traditional view of driving pacemaker function of thalamus), 2) feedforward and feedback inhibition of principal neurons via the intracortical interneuron network, and, eventually, 3) massive context-dependent and task-related top-down projections, for example, from motor cortex and various other frontal areas (Swadlow and Gusev 2001; Klimesch et al. 2007; Miller et al. 2007; Bollimunta et al. 2011; Neske et al. 2015). For instance, simulation of the above-mentioned actors in a columnar S1 model sufficiently reproduced magneto-encephalographic data on humans (Jones et al. 2009). In our study, the implementation of subthreshold stimulation serves as an experimental tool to selectively investigate the role of intracortical inhibitory interneuron networks in mu rhythm modulation. The imperceptibility of the stimulation and the task-free paradigm jointly ensure that afferent input processing does not entail conscious—in our case “confounding”—perception-related processes such as expectation, motor preparation and response, attentional focusing, or habituation, which is frequently observed in the context of supraliminal stimulation.

We demonstrate that subthreshold stimulation modulates mu-alpha activity; however, single pulses and pulse trains exert opposite effects: Confirming our previous findings (Nierhaus et al. 2015; Forschack et al. 2017), alpha synchronization is induced after consecutive single pulses, whereas desynchronization emerges after pulse trains. Alpha activity has been frequently linked to inhibition (Jensen and Mazaheri 2010; Klimesch et al. 2007). For the visual system, it has been shown that higher alpha power in posterior regions correlates with lower detection rates of visual stimuli (Ergenoglu et al. 2004; Babiloni et al. 2006; Hanslmayr et al. 2007; van Dijk et al. 2008). Similarly, for the somatosensory system, endogenous prestimulus alpha power has been shown to influence perception (Schubert et al. 2009). While these studies focus on the functional relevance of spontaneously fluctuating (endogenous) background rhythms, we have previously suggested that exogenous modulation of background rhythms might mediate the effect of subthreshold stimulation on target detection (Bareither et al. 2014b; Nierhaus et al. 2015). Following this notion, we expected detection impairment for a target stimulus delivered within the period of increased alpha power (~150 to ~250 ms) induced by subthreshold single pulse stimulation. However, no detection impairment was observed for any of the three delays tested.

Repetitive application of the same subthreshold stimulus no longer increases mu-alpha activity; instead, a long-lasting desynchronization is observed. Different from mere event-related desynchronization, consistently observed for suprathreshold stimulation (Neuper et al. 2006), we assume that during subthreshold train stimulation the induction of synchronization by the first subthreshold “single” pulse is conflicted by signal processing associated with the subsequent subthreshold pulses: Synchronization should occur ~ 200 ms after the initial subthreshold pulse. However, the succeeding intra-train stimuli at the pulse period of ~ 140 ms (7 Hz) prevent this process to evolve; instead, the repetitive input leads to mu-alpha desynchronization. Moreover, subthreshold train stimulation consistently leads to a decrease of TP detection regardless of the time delay to the preceding subthreshold pulse. Each tested delay falls into the period of desynchronization, but only the 180 ms is well beyond the decay phase of synaptic inhibition. Consequently, we regard train-induced detection decrease for late presented target stimuli as a convincing link between mu-alpha desynchronization and inhibitory interneuron activity. The desynchronization that we observe, however, is not equivalent to reduced alpha power as it occurs in endogenous alpha fluctuations, as discussed above. Rather, our results indicate that an altered state of cortical processing induced by the pulse train persists at least ~ 2 s beyond stimulation. Consistent with this notion, we find a divergent effect of single pulses after controlling for pre-trial history (i.e., hysteresis): By contrasting SaS versus SaT, we disclosed a significant difference between the single pulse trials depending on the previous trial stimulation. While SaT tends to decrease alpha synchrony, SaS seems rather associated with a transient mu-alpha increase similar to the one previously reported (Nierhaus et al. 2015; Forschack et al. 2017). Still, only the contrast of the two conditions was statistically significant but not the power change versus baseline. This may be due to low statistical power after trial sorting and thus have to be confirmed by future studies. It is known that representations of task- or stimulus-related information can persist in the absence of stimulation in patterns of synaptic weights (Jonides et al. 2008). The considerably long persistence of train-induced desynchronization together with the hysteresis effect suggests a persistent shift in cortical excitation-inhibition balance (Isaacson and Scanziani 2011), where also mechanisms in synaptic plasticity can play a role. This is why a single subthreshold pulse can still reinforce the fading desynchronization. By design, however, the stimulus history effects cannot be directly translated from the EEG to the behavioral experiments and any correlation or interpretation of the behavioral data with the EEG data should be taken with caution. Nevertheless, our approach of implementing the same stimuli in separate experiments allows one to bring the electrophysiological and behavioral correlates together, which in turn will help to design combined EEG/behavioral paradigms addressing more detailed questions.

In conclusion, a single subthreshold pulse elicits mu-alpha synchronization but is not sufficient to exert measurable functional inhibition. Its sole repetition, however, progressively shifts the cortical excitation-inhibition balance toward a robust cortical inhibitory state that persistently affects subsequent stimulus processing and is paralleled by prolonged mu-alpha desynchronization. Evidently, the inhibitory component associated with subthreshold stimulation becomes functionally relevant only through repetition. Beyond the established notion of increased alpha activity acting as a mediator of functional inhibition, we demonstrate that it is possible to generate a cortical state of increased inhibition accompanied by mu-alpha desynchronization. Our findings offer a new approach for controlled nonconscious, noncognitive, and attention-independent manipulation of cortical synchrony and excitability in opposite directions.

## Notes

We thank S. Stasch for her excellent technical assistance. *Conflict of Interest*: None declared.
